# Dosimetric effects of a repositioning head frame system and treatment planning system dose calculation accuracy

**DOI:** 10.1002/acm2.12456

**Published:** 2018-09-25

**Authors:** Carlos Ferrer, Concepción Huertas, Rodrigo Plaza, Zulima Aza, Eva Corredoira

**Affiliations:** ^1^ Department of Medical Physics and Radiation Protection H.U. La Paz Madrid Spain

**Keywords:** attenuation, buildup, Fraxion, surface dose, treatment planning

## Abstract

This work aims to study the effect on surface dose and dose distribution caused by the Elekta Fraxion cranial immobilization system. The effect of Fraxion inclusion in Elekta Monaco treatment planning system and its calculation accuracy is also checked. To study the dose attenuation, a cylindrical phantom was located over the Elekta Fraxion with an IBA CC13 ionization chamber placed in the central insert at the linac isocenter. Dose measurements at multiple gantry angles were performed for three open fields, 10 × 10 cm, 5 × 5 cm and other smaller 2 × 2 cm. Measured doses were compared with the ones calculated by Monaco. Surface dose and dose distribution in the buildup region were measured placing several Gafchromic Films EBT3 at linac CAX between the slabs of a RW3 phantom located over Fraxion and read using FilmQA Pro software. Measures were performed for two open field sizes and results were compared with Monaco calculations. Measurements show a 1% attenuation for 180° gantry angle but it can be as high as 3.4% (5 × 5 open field) for 150°/210° gantry angle, as with these angles the beam goes through the Fraxion's headrest twice. If Fraxion is not included in the calculation Monaco calculation can result in a 3% difference between measured and calculated doses, while with Fraxion in the calculation, the maximum difference is 0.9%. Fraxion increases 3.7 times the surface dose, which can be calculated by Monaco with a difference lower than 2%. Monaco also calculated correctly the PDD for both open fields (2%) when Fraxion is included in the calculation. This work shows that the attenuation varies with gantry angle. The inclusion of Fraxion in Monaco improves the calculation from 3% difference to 1% in the worst case. Furthermore, the surface dose increment and the dose in the buildup region are correctly calculated.

## 1 INTRODUCTION

In stereotactic radiosurgery (SRS) treatments, high radiation dose is delivered to a small target volume within the brain in a single or several fractions, normally up to five. This requires high accuracy and precision in patient positioning as well as in delivered dose calculation, to locate the target properly and achieve a high‐gradient dose distribution reducing the dosage of normal structures.^1^

Designed in the fifties by L. Leksell,^2^, ^3^, ^4^ the use of a rigid invasive stereotactic head frame fixed to the skull using fixation screws ensures submillimeter precision, that makes it the ideal choice for one single fraction treatments, proven effective for small brain tumors and brain metastases.^5^, ^6^ Nevertheless, for lesions larger than 3 cm in diameter, or when more than one lesion is involved, the surrounding normal tissue exposed to the single high dose augments, increasing the toxicity, as well as the risks of neurological morbidity from radiation necrosis and local treatment failure.^5^, ^7^, ^8^, ^9^, ^10^ For lesions larger than 3 cm, fractionated stereotactic radiotherapy (SRT) has been proved to be an effective and safe technique to treat brain metastases,^11^ as it delivers a high biologically effective dose with the advantage of the effects of fractionation on normal brain tissue, while being highly efficient achieving local tumor control.^12^

Fraxion (Elekta, A.B., Stockholm, Sweden) is the commercial name of the repositioning head frame system designed to overcome the drawbacks of conventional invasive fixation for linac‐based SRS, and SRT treatments. This repositioning system is based on the design of the Elekta Extend™ System for Leksell Gamma Knife™ (Elekta, A.B., Stockholm, Sweden), which has been described previously in the literature.^13^, ^14^

Although external devices to the patient are meant to be radio‐translucent, it is well‐known that these devices increase the skin dose^15^, ^16^ and alter the dose distribution. This is due to the beam attenuation as it crosses the device, which acts as a bolus and backscatter material. The skin sparing effect for megavoltage beams and depth of maximum dose decrease, hence increasing the skin dose.^17^, ^18^

The American Association of Physicists in Medicine (AAPM) Task Group 176 reported the dosimetric effects caused by different couch tops and immobilization devices.^19^ As Fraxion was not included among the devices incorporated in this report, the objective of this study was to measure the dose attenuation caused by the Fraxion headrest part for different gantry angles and field sizes, as this part is commonly traversed by the linac beams when the SRT treatments are planned with volumetric modulated arc therapy (VMAT) technique,^20^ particularly when more than one lesion is involved and sometimes a full arc is needed.^21^, ^22^ Also the variation in surface dose was measured. Finally, the dose calculation accuracy with Monte Carlo based Elekta Monaco treatment planning system (TPS) (v. 5.00.00) when Fraxion is included was checked, along with the clinical impact when it is not included.

## 2 MATERIALS AND METHODS

The Fraxion system basically consists of a patient control unit (PCU), and a Fraxion frame with headrest and front piece. It also includes a unique vacuum mouthpiece and a head vacuum cushion which fits into two holes on the bottom of the headrest to achieve accurate and comfortable patient immobilization, which combined with partial or full head thermoplastic masks ensures patient immobilization and positioning accuracy. The PCU provides the necessary vacuum for securing the mouthpiece to the patient's maxilla, thus securely immobilizing the patient. In addition, the PCU is used to form the vacuum cushion.*

**Figure 1:**
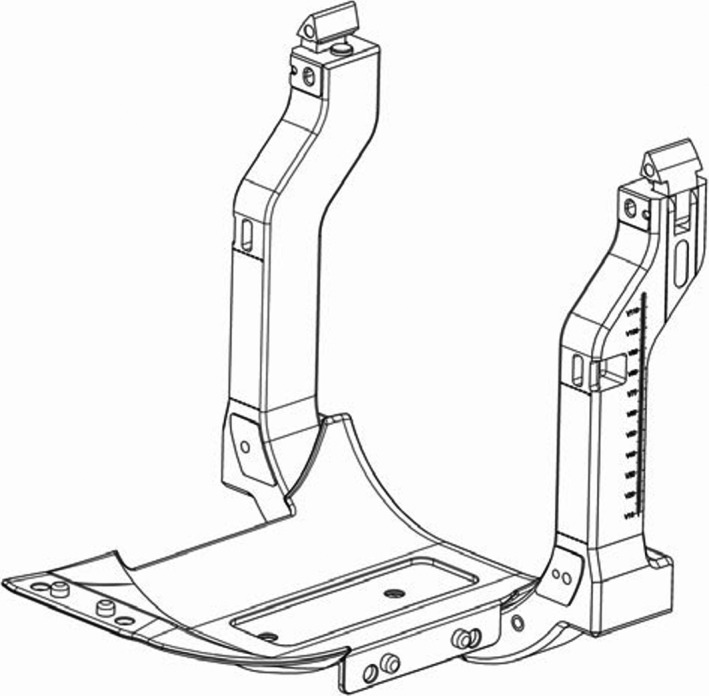
Illustration of the Fraxion's headrest and posts. Reproduced with permission from the vendor.

Figure 1 shows an illustration of the headrest and posts used to clamp the mouthpiece. These posts are made of a foam core sandwiched between carbon fiber, and are not included in our attenuation measurements, as they are not crossed by radiation beams even when treating lesions at the cerebellum tonsil.^23^ The headrest is a 2 mm thick carbon fiber piece, slightly curved, with extensions on both sides to attach the patient mask, through which we assume that the attenuation will be higher.

### 2.A Attenuation measurements

**Figure 2:**
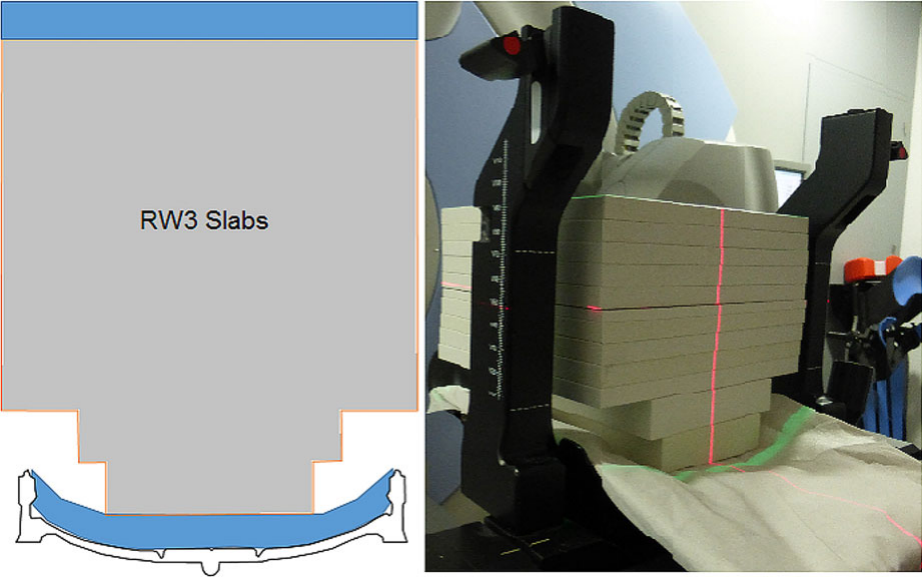
Cylindrical phantom placed over Fraxion's headrest for attenuation measurements.

Fraxion was placed on an Elekta Synergy linear accelerator equipped with a 160‐leaf Agility MLC and 6 MV beam energy. To study the dose attenuation a cylindrical PMAA phantom (15 cm diameter, 25.5 cm length, physical density 1.18 g/cm^3^) was located over the Fraxion with a 0.13 cm^3^ active volume IBA CC13 ionization chamber (IBA dosimetry GmbH, Schwarzenbruck, Germany) placed in the central insert at the linacisocenter (Fig. 2). The choice of this chamber is due to the fact that it provides good dose measurement results for the three field sizes used in this work, so it is not necessary to change it as the size of the field is modified.^24^, ^25^

Measurements performed at 0°, 90° and 270° gantry angles were used to obtain the reading without attenuation, as with these angles the beam doesn't traverse Fraxion. Reference dose without Fraxion was then the average dose at 0°, 90°, and 270°. 100 MU were delivered at each angle. These measurements were also used to verify dosimetrically the chamber position at the linacisocenter. Position was considered well centered once the readings differed ±0.1%, as described by Gerig et al.^26^ Dose attenuation measurements at various gantry angles were performed for three open fields, 10 × 10, 5 × 5 and 2 × 2 cm^2^. The gantry angles were the ones which cross Fraxion (135°–225°, 5°–10° increment, IEC gantry angles). The definitive measured values for each gantry angle were the average doses of symmetrical angles from 180° (180° ± *θ*°). All measurements were performed three times. The same measurements were calculated in Monaco with a 1.5 mm calculation grid and 1% statistical uncertainty, after acquiring the Fraxion and phantom images with the ionization chamber placed in the central insert, with a Toshiba Activion 16 CT (Toshiba Medical Systems, Ōtawara, Tochigi, Japan). Slices acquired were 1 × 1 and 512 × 512 mm^2^ (1 mm slices, 50 × 50 cm^2^ FOV, 120 KVp, 512 × 512 matrix). Attenuation was defined as:

$$\text{Attenuation}=\left(D_{0}-D_{F}\right)/D_{0}\times 100\left(\%\right)$$

similar to the one proposed by Smith et al.^27^, and where D_0_ is the dose measured without traversing Fraxion and D_F_ is the dose measured traversing Fraxion at different angles.

### 2.B Surface dose and buildup region

**Figure 3:**
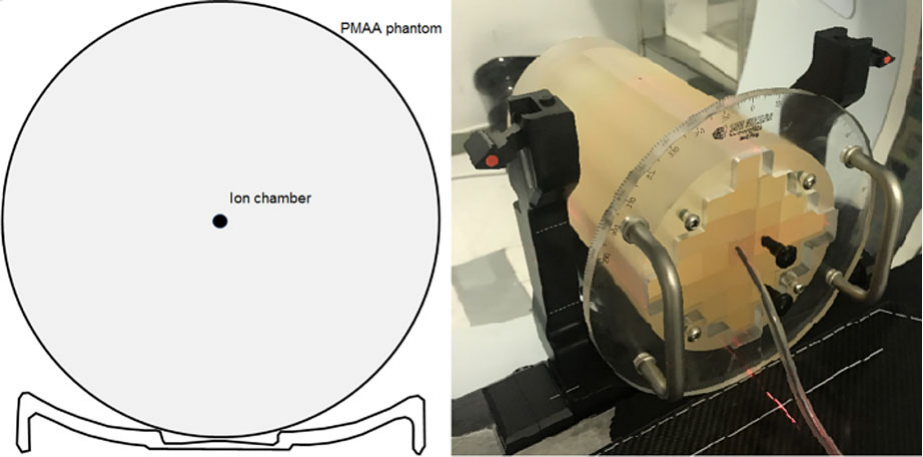
RW3 slabs phantom with radiochromic films placed between to measure the dose distribution.

To measure the surface dose and the dose distribution in the buildup region, another phantom was placed over Fraxion (*SSD* = 90 cm), using 30 × 30 cm^2^ RW3 slabs of 1 cm thickness (PTW Freiburg GmbH, Germany) and two additional 18 × 30 cm^2^ and 15 × 30 cm^2^ RW3 slabsof 2 cm thickness over 1.5 cm bolus to adapt the phantom to the Fraxion curved surface (Fig. 3). Another 1.5 cm bolus was added over the phantom to have symmetrical measures at 0° and 180° gantry angles. Several Gafchromic EBT3 radiochromic films (Ashland Advanced Materials, Niagara Falls, NJ, USA) were placed at linac CAX between the slabs at various depths. Films were situated at the surface, at 0.5 and 1.5 cm depths, and at the linac isocenter, and 200 MU were delivered for 10 × 10 and 5 × 5 cm^2^ open field sizes and 0° gantry angle. To ensure that films were placed parallel one to each other, they were stuck to the RW3 slabs, horizontally leveled with acalibrated digital level, or between bolus layers ensuring its correct placement. Once irradiated and removed, another set of films were placed under the phantom, in contact with Fraxion, and at 0.5, 1.5 cm from Fraxion, as well as the linac isocenter. Additional films were located 1 cm away from CAX as in this section Fraxion is slightly wider than in the middle, where the holes for the cushion are. Same field sizes and MU with 180° gantry angle were employed. This waywe can compare the effect of Fraxion in the buildup region as well as in the skin and surface dose using the formula:

$$\text{Variation}=-\left(D_{0,z}-D_{180,z}\right)/D_{0,z}\times 100$$

where D_0,z_ is the measured dose with gantry at 0 degrees and z (cm) depth, and D_180,z_ is the measured dose at the same relative position with the gantry at 180 degrees and z (cm) depth.Using this configuration, for both gantry angles, measurements are made at the same depths.

Films were kept in a light‐proof container when not being analyzed to reduce coloration from ambient light and UV sources^28^and handled according to the procedures described in the AAPM TG‐55 report.^29^ Two hours after irradiation films were scanned five consecutive times in transmission mode using an Epson Expression 11000 XL scanner, placing each piece of film one by one in portrait direction at the center of the scanner to prevent nonuniformity response effects. Scans were performed at 72 dpi resolution and analyzed with FilmQA Pro software (Ashland Advanced Materials, Niagara Falls, NJ, USA). Dose values presented in this work were the average values obtained for the five scans using the red channel and a previous film batch calibration. A 1 × 1 cm^2^ region of interest was selected in the middle of each film to obtain these values. The standard deviation in dose values normalized to the maximum is 0.5%, which results for a dose level of 200 cGy to average variations of ±1 cGy. These values agree with others reported in the literature.^24^

This phantom was also CT scanned with the same technique as the previous one, and images were exported to Monaco planning system where its external surface was contoured. The same measurements made with film were reproduced in Monaco to check the dose accuracy calculation at surface, 0.5, 1.5, and 10 cm (isocenter) depths compared with the values obtained with film. The variation in surface and skin dose with and without Fraxion was also calculated by Monaco, in the same way as was done previously with film and also removing Fraxion from the calculation to check that the attenuation calculated by Monaco was the same using:

$$\text{Attenuation}=\left(D_{\mathrm{WF}}/D_{F}\right)/D_{\mathrm{WF}}\times 100\left(\%\right)$$

where D_WF_ is the calculated dose without Fraxion and D_F_ is the calculated dose with Fraxion included in the calculation.

## 3 RESULTS

### 3.A Attenuation measurements

Table 1 shows the percentage transmission/attenuation measurement results as a function of the gantry angle for the three field sizes considered, 2 × 2, 5 × 5 and 10 × 10 cm^2^ and 6 MV beam energy. The attenuation at each gantry angle is calculated relative to the average reading at 0, 90 and 270 degrees. Transmission is the result of applying the formula: %*T* = 100 − %attenuation.

**Table 1:** Transmission/attenuation results

Angle (°)	Field size 2 × 2 cm^2^			Field size 5 × 5 cm^2^			Field size 10 × 10 cm^2^		
	cGy	T (%)	Att (%)	cGy	T (%)	Att (%)	cGy	T (%)	Att (%)
0	73.18	100.00	0.00	81.05	100.00	0.00	88.00	100.00	0.00
90									
270									
135	73.15	99.96	0.05	80.99	99.92	0.08	87.99	99.99	0.01
140	73.17	99.98	0.02	80.98	99.91	0.09	88.01	100.01	‐0.01
150	70.78	96.72	3.28	78.32	96.63	3.37	85.24	96.86	3.14
160	71.93	98.29	1.71	79.44	98.01	1.99	86.47	98.26	1.74
170	72.23	98.69	1.31	79.80	98.45	1.55	86.84	98.68	1.32
180	72.43	98.97	1.03	80.04	98.75	1.25	87.11	98.99	1.01

**Figure 4:**
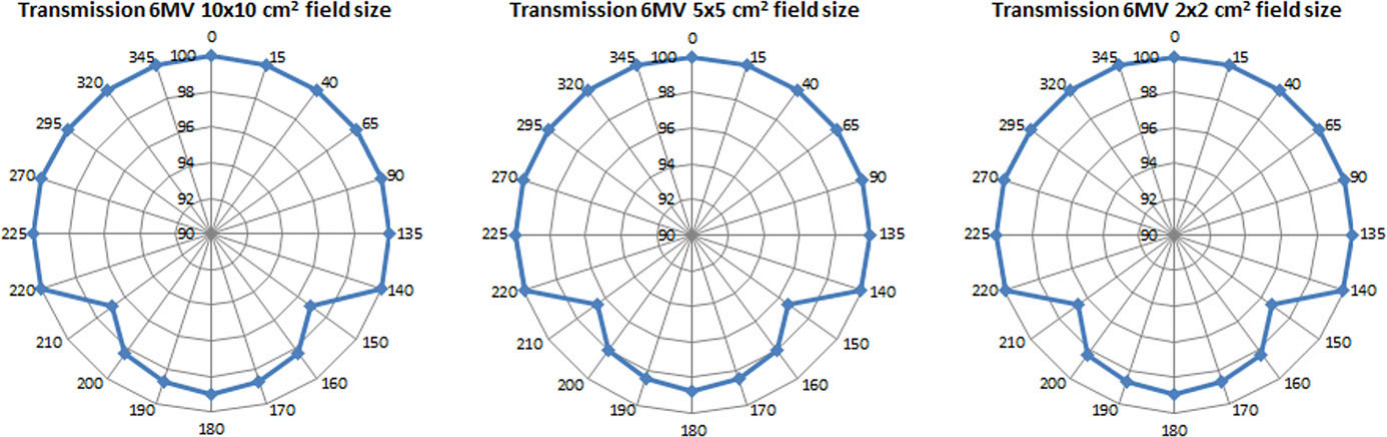
Percentage transmission for 10 × 10 and 5 × 5 cm^2^ field size as a function of the gantry angle.

It can be seen that attenuation is greater at 150° and 210° gantry angles as expected, since the beam crosses twice the Fraxion structure. At these angles, attenuation can be as high as 3.4% for 5 × 5 cm^2^ field size and decreases slightly with increasing field size from 5 × 5 to 10 × 10 cm^2^, but this tendency reverses for 2 × 2 cm^2^ field size. Attenuation results in approximately 1% for 180° gantry angle as stated in the Fraxion manual provided by Elekta. Figure 4 shows the percentage transmission as a function of gantry angle for the three field sizes and 6 MV beam energy.

As explained before, each data point is the average of symmetrical measurements around 180° (180° ± *θ*°) due to the left–right symmetry and the central position of the chamber and the cylindrical phantom over Fraxion.

### 3.B Attenuation therapy planning system calculation

**Figure 5:**
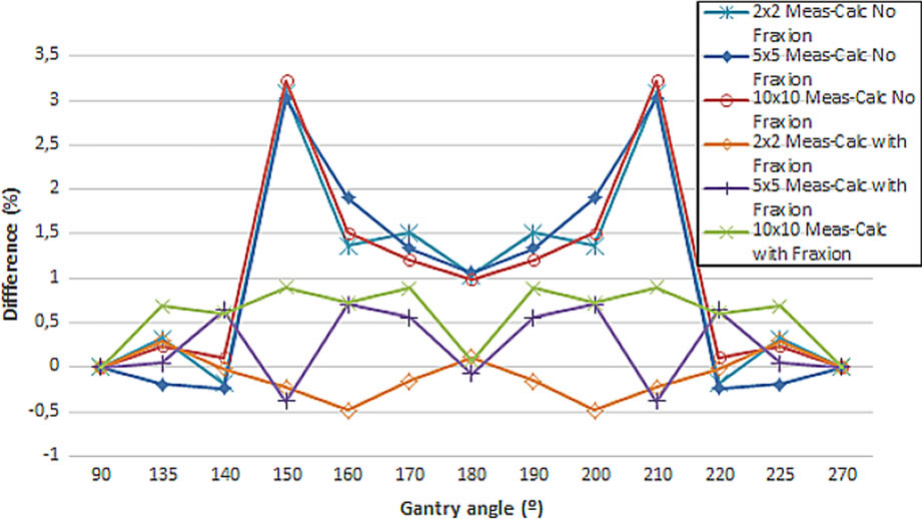
Difference between measured and calculated percentage attenuation when Fraxion is included or not in the calculation for the three field sizes.

The percentage attenuation calculated with Monaco was carried out using the TC images acquired with the parameters mentioned previously. Two calculations were performed, one without Fraxion in the calculation, and the other with Fraxion included in the calculation. Figure 5 represents the difference between the measured and the calculated attenuation. If Fraxion is not included in the calculation, the difference between measured and calculated attenuation can result as high as 3%, while with Fraxion in the calculation, the maximum difference is 0.9% for 10 × 10 cm^2^ field size and corresponding to the gantry angles for which the attenuation is higher, 150° and 210°. For 5 × 5 cm^2^ field size, the maximum difference between measured and calculated attenuation resulted 0.7% for 160° and 220° gantry angles, through which the beam also traverses twice Fraxion. For the smaller measured field, 2 × 2 cm^2^, differences obtained are even smaller, 0.5% for 160° and 220° gantry angles.

Data shown in Fig. 5 is only valid when Fraxion is centered laterally as attenuation is a function of the path traversed by the beam through Fraxion, as observed in other works^26^, ^30^and probably is the reason why the maximum attenuation difference between measured and calculated values are not coincident between the two field sizes included in this study.

### 3.C Surface dose and buildup region

**Figure 6:**
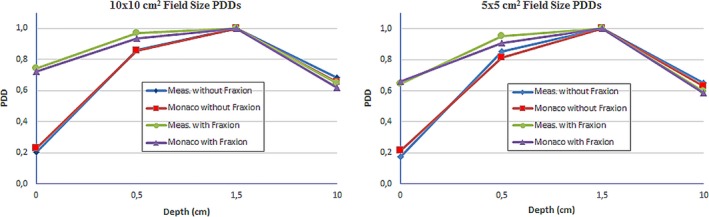
Measured and calculated PDD curves for 10 × 10 and 5 × 5 cm^2^ field sizes, with and without Fraxion.

The presence of Fraxion during the treatment is going to alter the dose, particularly affecting the surface, skin and buildup region doses. Figure 6 shows the measured and calculated Percentage Depth Dose (PDD) curves when Fraxion is included, and when it's not.

Measures include the surface dose, dose in the buildup region, maximum dose and dose at the isocenter, at 10 cm depth. Maximum PDD differences between measured and calculated values are 4% at surface without Fraxion and at 0.5 cm depth with Fraxion for 5 × 5 cm^2^ field size. For 10 × 10 cm^2^ field size, these differences are 2% at the surface and 3% at 0.5 and 10 cm depth with Fraxion. These differences correspond to measures made under the same conditions, with measured and calculated doses, both with Fraxion or without Fraxion included. In this way, it is possible to check that Monaco calculates the PDD correctly, below 4% difference for 5 × 5 cm^2^ field size, and less than 2% when Fraxion is included in the calculation. At the isocenter, with Fraxion included in the calculation, Monaco underestimates the dose by 3% for 10 × 10 cm^2^ field size, but only by 1% for 5 × 5 cm^2^ field size.

Table 2 summarizes the percentage (to maximum dose) surface dose results. Measured values show that Fraxion increments 3.6 times the surface dose for 10 × 10 cm^2^ field size and by 3.7 times for 5 × 5 cm^2^ field size. This result is similar for calculated values. The last column shows the difference obtained if Fraxion is included or not in the calculation [calculated surface dose (%) with Fraxion minuscalculated surface dose (%) without Fraxion]. If Fraxion is not included in the calculation, percentage differences between percentage surface dose values can be as high as 50% for 10 × 10 cm^2^ or 45% for 5 × 5 cm^2^ field sizes. These differences are 3% larger with measured values, which proves the ability of the Monaco algorithm to calculate the dose.

**Table 2:** Difference between measured and calculated surface dose, and difference when Fraxion is included or not in the calculation

Field size (cm^2^)	Fraxion	Surface dose (% of max)			Difference calculated with/without fraxion (%)
		Measured (%)	Calculated (%)	Difference measured/calulated (%)	
10 × 10	Without fraxion	20.4	22.6	2.1	49.7
	With fraxion	74.3	72.2	−2.1	
5 × 5	Without fraxion	17.4	21.4	4.0	44.6
	With fraxion	64.5	66.0	1.5	

**Figure 7:**
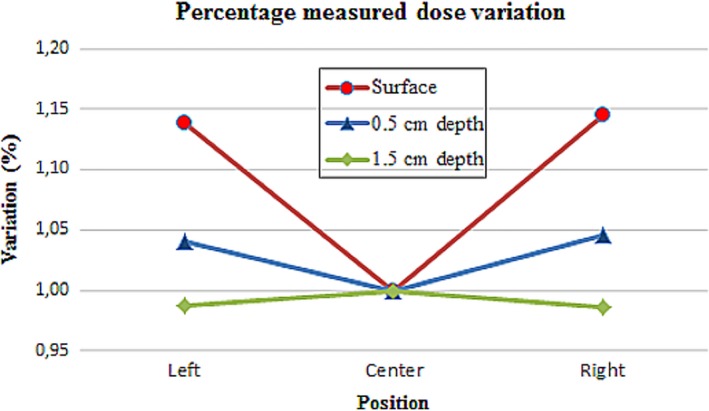
Percentage dose variation due to the different thickness of Fraxion headrest at surface and two other depths.

The variation in the headrest thickness due to the holes used to fit the patient cushion in Fraxion was also studied. Figure 7 represents the percentage variation in the measured dose, normalized to the dose at the center of Fraxion.

Due to the different thickness, the buildup effect is easily confirmed. Differences between doses measured at the center and the sides present a higher value at the surface than the ones measured at 0.5 cm depth. At 1.5 cm depth this effect is no longer present and the dose value is smaller, due to the attenuation caused by Fraxion.

## 4 DISCUSSION

As indicated in the AAPM report TG‐176 and by several other authors,^18^, ^19^, ^20^, ^26^, ^27^, ^30^, ^31^, ^32^ devices external to the patient can act as a bolus, increase the skin dose, attenuate the beam and in summary modify the dose distribution. This effect can be observed even with a vacuum cushion placed in the beam's path.^33^ Although this effect is well‐known, no previous studies included the attenuation of Fraxion's headrest for different gantry angles. In this work, the dose attenuation effects caused by Fraxion's headrest have been studied. Several gantry angles were studied, especially the ones for which the beam traverses the structure twice, where the patient's head mask is clamped.

Modern commercially available planning systems have the option to include the treatment couch, as Monaco does. Its inclusion in the treatment plan is nowadays a common practice, but in some cases this can be missed when attenuation is expected to be negligible. It has also been studied, how well Monaco is capable of calculating the dose at the surface, the buildup region, and the isocenter when Fraxion is included in the calculation, without any other data modification, that is, without forcing any Fraxion density, only with the one obtained from the CT images.

Results show that attenuation at 180° gantry angle is approximately 1%, measured at the isocenter, which coincides with the data provided by the vendor. For the three field sizes included in this work, the maximum attenuation value is obtained for 150° gantry angle, for which attenuation can be as high as 3%, and decrease as the gantry angle approaches 180°. At approximately 150°, the beam traverses the headrest part where the patient's mask is clamped, the structure folds and the beam passes through twice. As expected, for the three field sizes, the more radiological path travelled by the beam the more attenuation value obtained. So although carbon fiber materials are considered radio transparent to high energy photons,^34^, ^35^ the results obtained in this work show that the attenuation caused by Fraxion's headrest can be of importance when planning SRS and SRT treatments with VMAT technique, with which arcs traversing the structure can be used.

There is a slight dependence of the attenuation with the field size, it decreases as the field size increases for 5 × 5 and 10 × 10 cm^2^, but increases with field size from 2 × 2 to 5 × 5 cm^2^. Attenuation of the 6 MV photon beam at 150° gantry angle decreases from 3.4% to 3.1% for 5 × 5 and 10 × 10 cm^2^ field sizes, respectively, but increases from 3.3% to 3.4% for 2 × 2 and 5 × 5 cm^2^ field sizes, respectively. The latter is in contrast with other authors that studied attenuation effects of couch tables.^30^, ^36^, ^37^ For a smaller field size, Njeh et al.^37^ figured that scatter radiation which is recorded by the chamber is reduced, and this resulted in the decrease in attenuation as the field size increased, which occurs between 5 × 5 and 10 × 10 cm^2^ field sizes but not between 2 × 2 and 5 × 5 cm^2^ field sizes. The difference between their study and this one is the shape of the headrest. Fraxion's headrest shape is slightly curved, with different thickness at its bottom, and folds where the patient's mask is clamped. At this headrest part, the beam traverses twice the structure and the attenuation results turn out to be higher. For a 2 × 2 cm^2^ field size, the beam area which traverses twice the structure at 150° gantry angle is less than for a 5 × 5 cm^2^ field size, and due to that, the attenuation is smaller than for 5 × 5 cm^2^ field size, but greater than for 10 × 10 cm^2^ field size.

**Figure 8:**
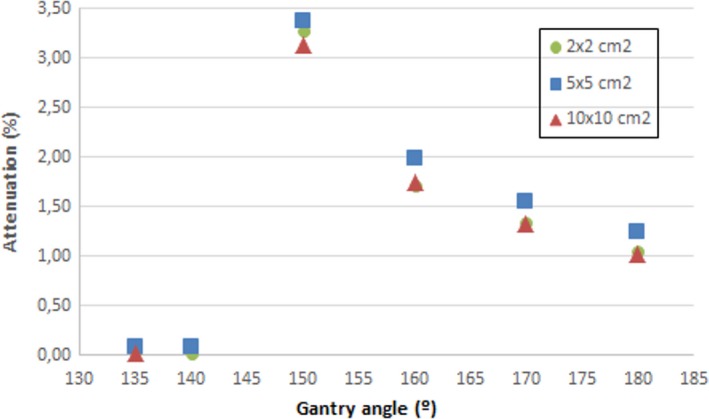
Attenuation variation with gantry angle for three field sizes.

This variation in attenuation as the field size variesis also dependent on the gantry angle. Figure 8 shows the headrest attenuation (%) for 2 × 2 cm^2^, 5 × 5 cm^2^ and 10 × 10 cm^2^ field sizes. Attenuation is negligible for135° and 140°, as the beam does not traverse Fraxion's headrest, but it is more than 3% where the beam traverses the structure twice. At 160°, attenuation approaches to its 1% value at 180° as part of the beam crosses the structure again only once. This value is not 1% as stated on the Fraxion manual as part of the beam is still going through the structure twice and because of the different thickness at the headrest bottom. Although its shape is slightly curved, its curvature is not concentric with the gantry movement, which causes variations in the radiological path traveled through the beam.

Skin toxicity has always been a major concern in radiotherapy treatments, even nowadays with modern techniques.^38^, ^39^, ^40^ As discussed earlier, surface dose and dose at 0.5 cm depth have been measured. Results show the important effect caused by the headrest on skin dose, as the measured dose increases significantly at both depths. The effect on surface dose caused by the different headrest thickness at its bottom is also proved. Surface dose is 15% higher on the sides of the headrest than at its center, due to the buildup effect. This effect fades with the beam depth in the phantom, but affects to the skin dose in those areas.

The inclusion of Fraxion or any other immobilization device in Monaco treatment plan is straightforward. It has been demonstrated that Monaco correctly calculates the dose when the headrest is included in the calculation. Attenuation at different gantry angles is calculated with less than 2% difference from measured values.PDD are calculated with less than 3% difference with measured values, which is in accordance with other authors who employed Monte Carlo based TPS,^41^ although differences in surface dose increase as the field sizes decreases, so probably it's better to avoid high attenuation areas when planning.

**Figure 9:**
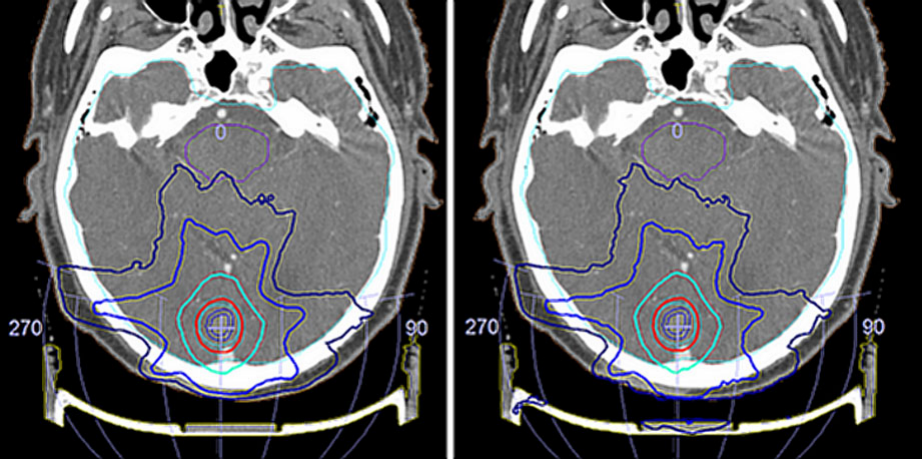
Treatment differences when Fraxion is not included in the calculation (left) or when it is (right).

Figure 9 shows the variation in the calculation when Fraxion is included or not, when planning an SRS treatment with seven non‐coplanar arcs prescribed at 18 Gy in one single fraxion. In this case, the treatment was planned without Fraxion. Then it was included and exactly the same plan was again calculated, to see the “real” dose distribution during treatment. It can be seen that if Fraxion is not included, its effect at the surface and hence the skin dose is missed. Also, dose to the target above 20 Gy resulted in *V*_20_ = 52.3% if Fraxion is not considered, and only 0.08% when it is. Therefore, it is possible to get wrong dose values that can be of importance when Fraxion is ignored. This causes a direct clinical impact on the patient, as the PTV will receive in general, fewer doses than the ones prescribed, leading to a mistreatment of the tumor. Besides, the information given by the planning system about the doses received by the OARs will be incorrect. Dose tolerances could be exceeded, causing fatal radiation injuries to the patient. These considerations prove the importance of Fraxion inclusion in the treatment planning.

## 5 CONCLUSIONS

The effect on attenuation of Fraxion headrest for 6 MV has been studied. Although attenuation is less than 2% for several gantry angles, is more than 3% where the headrest folds to clamp the patient mask. This can be of importance if Fraxion is not included in the calculation when the beam traverses that part with complete VMAT arcs or non‐coplanar arcs, which coincide with that area. Monaco correctly calculates the attenuation and the PDD, but differences in surface dose are greater than 5% for small fields, so it is better to avoid the maximum attenuation area when planning radiotherapy treatments. Results show the importance of the inclusion of Fraxion in the treatment planning, and the accuracy of the calculation when it is included without any density manipulation in the TPS.

## CONFLICTS OF INTEREST

The authors have no relevant conflicts of interest to disclose.

## NOTE

1 Revision E. Fraxion™ User Manual. Medical Intelligence Medizintechnik GmbH. Schwabmünchen. Germany. 2013.

## References

[1] Stanley J , Breitman K , Dunscombe P , Spencer DP , Lau H . Evaluation of stereotactic radiosurgery conformity indices for 170 target volumes in patients with brain metastases. J Appl Clin Med Phys. 2011;12:245–253. 10.1120/jacmp.v12i2.3449 PMC5718686 21587193

[2] Leksell L . The stereotaxic method and radiosurgery of the brain. Acta Chir Scand. 1951;102:312–319. 14914373

[3] Leksell L . Occasional review stereotactic radiosurgery. J Neurol Neurosurg Psychiatry. 1983;46:797–803. 6352865 10.1136/jnnp.46.9.797 PMC1027560

[4] Leksell L , Lindquist C , Adler JR , Leksell D , Jernberg B , Steiner L . A new fixation device for the Leksell stereotaxic system. Technical note. J Neurosurg. 1987;66:626–629. 3549998 10.3171/jns.1987.66.4.0626

[5] Shaw E , Scott C , Souhami L , et al. Single dose radiosurgical treatment of recurrent previously irradiated primary brain tumors and brain metastases: final report of RTOG protocol 90‐05. Int J Radiat Oncol Biol Phys. 2000;47:291–298. 10802351 10.1016/s0360-3016(99)00507-6

[6] Alexander E , Moriarty TM , Davis RB , et al. Stereotactic radiosurgery for the definitive, noninvasive treatment of brain metastases. J Natl Cancer Inst. 1995;87:34–40. 7666461 10.1093/jnci/87.1.34

[7] Blonigen BJ , Steinmetz RD , Levin L , Lamba MA , Warnick RE , Breneman JC . Irradiated volume as a predictor of brain radionecrosis after linear accelerator stereotactic radiosurgery. Int J Radiat Oncol Biol Phys. 2010;77:996–1001. 19783374 10.1016/j.ijrobp.2009.06.006

[8] Minniti G , Clarke E , Lanzetta G , et al. Stereotactic radiosurgery for brain metastases: analysis of outcome and risk of brain radionecrosis. Radiat Oncol. 2011;6:48. 21575163 10.1186/1748-717X-6-48 PMC3108308

[9] Jairam V , Chiang VLS , Yu JB , Knisely JPS . Role of stereotactic radiosurgery in patients with more than four brain metastases. CNS Oncol. 2013;2:181–193. 24273642 10.2217/cns.13.4 PMC3835313

[10] Li X , Li Y , Cao Y , et al. Safety and efficacy of fractionated stereotactic radiotherapy and stereotactic radiosurgery for treatment of pituitary adenomas: a systematic review and meta‐analysis. J Neurol Sci. 2017;372:110–116. 28017195 10.1016/j.jns.2016.11.024

[11] Jeong WJ , Park JH , Lee EJ , Kim JH , Kim CJ , Cho YH . Efficacy and safety of fractionated stereotactic radiosurgery for large brain metastases. J Korean Neurosurg Soc. 2015;58:217–224. 26539264 10.3340/jkns.2015.58.3.217 PMC4630352

[12] Song CW , Park H , Griffin RJ , Levitt SH . Radiobiology of stereotactic radiosurgery and stereotactic body radiation therapy In: Technical Basis of Radiation Therapy. Berlin: Springer; 2012 10.1007/174_2011_264

[13] Ruschin M , Nayebi N , Carlsson P , et al. Performance of a novel repositioning head frame for gamma knife perfexion and image‐guided linac‐based intracranial stereotactic radiotherapy. Int J Radiat Oncol Biol Phys. 2010;78:306–313. 20385456 10.1016/j.ijrobp.2009.11.001

[14] Sayer FT , Sherman JH , Yen CP , Schlesinger DJ , Kersh R , Sheehan JP . Initial experience with the eXtend System: a relocatable frame system for multiple‐session Gamma Knife radiosurgery. World Neurosurg. 2011;75:665–672. 21704934 10.1016/j.wneu.2010.12.051

[15] Butson MJ , Cheung T , Yu PKN . Megavoltage x‐ray skin dose variation with an angle using grid carbon fibre couch tops. Phys Med Biol. 2007;52:N485–N492. 17921572 10.1088/0031-9155/52/20/N03

[16] Connor M , Wei RL , Yu S , Sehgal V , Klempner SJ , Daroui P . Radiation dermatitis caused by a bolus effect from an abdominal compression device. Med Dosim. 2016;41:221–224. 27264694 10.1016/j.meddos.2016.02.003

[17] Butson MJ , Cheung T , Yu PKN , Webb B . Variations in skin dose associated with linac bed material at 6 MV x‐ray energy. Phys Med Biol. 2002;47:N25–N30. 11820224 10.1088/0031-9155/47/1/404

[18] Poppe B , Chofor N , Rühmann A , et al. The effect of a carbon‐fiber couch on the depth‐dose curves and transmission properties for megavoltage photon beams. Strahlentherapie und Onkol. 2007;183:43–48. 10.1007/s00066-007-1582-8 17225945

[19] Olch AJ , Gerig L , Li H , Mihaylov I , Morgan A . Dosimetric effects caused by couch tops and immobilization devices: report of AAPM Task Group 176. Med Phys. 2014;41:061501. 24877795 10.1118/1.4876299

[20] Ferrer C , Huertas C , Castaño A , Colmenar A , Mañas A , Serrada A . Dose conformation evaluation of volumetric modulated arc therapy for cranial radiosurgery. Radiother Oncol. 2016;119:S716.

[21] Salkeld AL , Unicomb K , Hayden AJ , Van Tilburg K , Yau S , Tiver K . Dosimetric comparison of volumetric modulated arc therapy and linear accelerator‐based radiosurgery for the treatment of one to four brain metastases. J Med Imaging Radiat Oncol. 2014;58:722–728. 24913419 10.1111/1754-9485.12188

[22] Iwai Y , Ozawa S , Ageishi T , Pellegrini R , Yoda K . Feasibility of single‐isocenter, multi‐arc non‐coplanar volumetric modulated arc therapy for multiple brain tumors using a linear accelerator with a 160‐leaf multileaf collimator: a phantom study. J Radiat Res. 2014;55:1015–1020. 24944266 10.1093/jrr/rru042 PMC4202300

[23] Ferrer C , Huertas C , Castaño A , et al. EP‐1531: collimator angle influence on dose coverage for VMAT SRS treatment of four brain metastases. Radiother Oncol. 2017;123:S823.

[24] Azangwe G , Grochowska P , Georg D , et al. Detector to detector corrections: a comprehensive experimental study of detector specific correction factors for beam output measurements for small radiotherapy beams. Med Phys. 2014;41:072103. 24989398 10.1118/1.4883795

[25] Godwin GA , Simpson JB , Mugabe KV . Characterization of a dynamic multi‐leaf collimator for stereotactic radiotherapy applications. Phys Med Biol. 2012;57:4643–4654. 22750675 10.1088/0031-9155/57/14/4643

[26] Gerig LH , Niedbala M , Nyiri BJ . Dose perturbations by two carbon fiber treatment couches and the ability of a commercial treatment planning system to predict these effects. Med Phys. 2010;37:322–328. 20175495 10.1118/1.3271364

[27] Smith DW , Christophides D , Dean C , Naisbit M , Mason J , Morgan A . Dosimetric characterization of the iBEAM evo carbon fiber couch for radiotherapy. Med Phys. 2010;37:3595–3606. 20831067 10.1118/1.3451114

[28] Alnawaf H , Yu PKN , Butson M . Comparison of Epson scanner quality for radiochromic film evaluation. J Appl Clin Med Phys. 2012;13:314–321. 10.1120/jacmp.v13i5.3957 PMC5718226 22955661

[29] Niroomand‐Rad A , Blackwell CR , Coursey BM , et al. Radiochromic film dosimetry: recommendation of AAPM radiation therapy committe task group 55. Med Phys. 1998;25:2093–2115. 9829234 10.1118/1.598407

[30] Nasseri S , Gholamhosseinian H , Momennezhad M , Naji M , Niazi S . The measurement of the beam attenuation and variation in skin dose due to the treatment couch in megavoltage radiotherapy. Int J Adv Inf Sci Technol. 2016;54:36–40. 10.15693/ijaist/2016.v54i54.36-40

[31] Huertas C , Ferrer C , Huerga C , Mas I , Serrada A . Treatment couch modeling in Elekta Monaco treatment planning system. Radiother Oncol. 2016;119:S389–S390.

[32] Seppälä JKH , Kulmala JAJ . Increased beam attenuation and surface dose by different couch inserts of treatment tables used in megavoltage radiotherapy. J Appl Clin Med Phys. 2011;12:15–23. 10.1120/jacmp.v12i4.3554. PMC5718753 22089010

[33] Takakura T , Ito Y , Higashikawa A , Nishiyama T , Sakamoto T . Verification of the dose attenuation of a newly developed vacuum cushion for intensity‐modulated radiation therapy of prostate cancer. Radiol Phys Technol. 2016;9:270–276. 27260347 10.1007/s12194-016-0359-0

[34] de Mooy LG . The use of carbon fibres in radiotherapy. Radiother Oncol. 1991;22:140–142. 1957004 10.1016/0167-8140(91)90010-e

[35] Meara SJP , Langmack KA . An investigation into the use of carbon fibre for megavoltage radiotherapy applications. Phys Med Biol. 1998;43:1359–1366. 9623664 10.1088/0031-9155/43/5/025

[36] Myint WK , Niedbala M , Wilkins D , Gerig LH . Investigating treatment dose error due to beam attenuation by a carbon fiber tabletop. J Appl Clin Med Phys. 2006;7:21–27. 10.1120/jacmp.v7i3.2247 PMC5722426 17533341

[37] Njeh CF , Raines TW , Saunders MW . Determination of the photon beam attenuation by the BrainLAB imaging couch: angular and field size dependence. J Appl Clin Med Phys. 2009;10:2979. 19692980 10.1120/jacmp.v10i3.2979 PMC5720553

[38] Penoncello GP , Ding GX . Skin dose differences between intensity‐modulated radiation therapy and volumetric‐modulated arc therapy and between boost and integrated treatment regimens for treating head and neck and other cancer sites in patients. Med Dosim. 2016;41:80–86. 26764180 10.1016/j.meddos.2015.09.001

[39] Cho GA , Ralston A , Tin MM , et al. In vivo and phantom measurements versus Eclipse TPS prediction of near surface dose for SBRT treatments. J Phys Conf Ser. 2014;489:012008.

[40] Hoppe BS , Laser B , Kowalski AV , et al. Acute skin toxicity following stereotactic body radiation therapy for stage I non‐small‐cell lung cancer: who's at risk? Int J Radiat Oncol Biol Phys. 2008;72:1283–1286. 19028267 10.1016/j.ijrobp.2008.08.036

[41] Heath E , Seuntjens J , Sheikh‐Bagheri D . Dosimetric evaluation of the clinical implementation of the first commercial IMRT Monte Carlo treatment planning system at 6 MV. Med Phys. 2004;37:540–549. 10.1118/1.1786172 15543782

